# How Many Days are Necessary to Represent Typical Daily Leg Movement Behavior for Infants at Risk of Developmental Disabilities?

**DOI:** 10.3390/s20185344

**Published:** 2020-09-18

**Authors:** Weiyang Deng, Ryota Nishiyori, Douglas L. Vanderbilt, Beth A. Smith

**Affiliations:** 1 Division of Biokinesiology and Physical Therapy, Herman Ostrow School of Dentistry, University of Southern California, Los Angeles, CA 90033, USA; 2 Children’s Hospital Los Angeles, Los Angeles, CA 90027, USA; rnishiyori@chla.usc.edu (R.N.); bsmith@chla.usc.edu (B.A.S.); 3 Department of Pediatrics, Keck School of Medicine, University of Southern California, Los Angeles, CA 90027, USA; DVanderbilt@chla.usc.edu

**Keywords:** infant, at risk, developmental disabilities, wearable sensor, minimum wear time

## Abstract

Background: Movement characteristics can differentiate between infants at risk and infants with typical development. However, it is unknown how many days are needed to accurately represent typical daily behavior for infants at risk of developmental disabilities when using wearable sensors. To consider the balance between participant burden and the amount of data collected and optimizing the efficiency of data collection, our study determined (1) how many days were necessary to represent typical movement behavior for infants at risk of developmental disabilities and (2) whether movement behavior was different on weekend days and weekdays. Methods: We used Opal wearable sensors to collect at least 5 days of 11 infants’ leg movement data. The standard (average of 5 days) was compared with four methods (average of the first 1/2/3/4 days) using the Bland–Altman plots and the Spearman correlation coefficient. We also compared the data from the average of 2 weekend days to the average of the first 2 weekdays for 8 infants. Results: The Spearman correlation coefficient comparing the average of the first 2 days of data and the standards were all above 0.7. The absolute differences between them were all below 10% of the standards. The Bland–Altman plots showed more than 90% of the data points comparing the average of 2 days and the standards fell into the limit of agreement for each variable. The absolute difference between weekend days and weekdays for the leg movement rate, duration, average acceleration, and peak acceleration was 15.2%, 1.7%, 6.8% and 6.3% of the corresponding standard, respectively. Conclusion: Our results suggest 2 days is the optimal amount of data to represent typical daily leg movement behavior of infants at risk of developmental disabilities while minimizing participant burden. Further, leg movement behavior did not differ distinctly across weekend days and weekdays. These results provide supportive evidence for an efficient amount of data collections when using wearable sensors to evaluate movement behavior in infants at risk of developmental disabilities.

## 1. Introduction

Nearly 1 in 6 children in the United States have a developmental disability and the rate has been increasing for the past decade [1]. Early diagnosis of developmental disabilities and identifying abnormal movement behavior patterns is important for this age group in order to provide appropriate early intervention [2]. Previous studies have found infants with or at risk of developmental disabilities show different movement characteristics compared to infants with typical development. For example, Ulrich and Ulrich found that infants with Down syndrome have less complex kicking patterns than infants with typical development [3]. Similarly, infants born preterm show slower acceleration, jerkier movement and lower variability of interlimb correlation than infants born full-term [4,5,6]. Infants with myelomeningocele demonstrated movements with more regular, repeatable patterns, shorter durations, and fewer acceleration peaks than infants with typical development [7,8].

Within the past decade, various measurement methods have been designed and validated for human movement using wearable technologies [9,10]. To accurately identify abnormal movement characteristics for infants with or at risk of developmental disabilities, we specifically developed and validated algorithms to analyze infant movement behavior using wearable sensors [11,12,13]. Compared to the traditional laboratory-based assessment with 3-dimensional motion capture systems, wearable technologies are less expensive, more portable and are able to collect consecutive data for hours or even days, which makes them more friendly for use in a home environment [14,15]. These factors are especially important for pediatric research if we want to understand what typical behavior is in a natural home environment. We believe using the wearable technologies at home will allow researchers and clinicians to collect longer periods and greater amounts of data than possible with previous technologies.

Wearable technologies (such as ActiGraph, Actiwatch, and Opal sensors) have been used to identify abnormal movement characteristics or provide guidance for treatment for infants with or at risk of developmental disabilities, such as in Down syndrome [16,17], cerebral palsy [18,19,20,21,22], autism spectrum disorder [23,24], Prader–Willi syndrome [25], attention-deficit/hyperactivity disorder [26], general developmental disabilities [27], and infants from high risk follow-up clinics [28]. Researchers have been trying to optimize the methods and protocols for using these technologies in order to generate more accurate and meaningful results for these technologies in infants with typical development [14,29,30,31,32]. However, most of these studies focus on children above 2 years of age or teenagers. In our previous work with infants with typical development, we found two days of data collection are optimal to accurately represent their typical daily behavior [14]. However, none of the existing methodological studies have included infants with or at risk of developmental disabilities.

Considering the atypical movement characteristics within this group, conclusions based on infants with typical development may or may not apply. Infants with or at risk for developmental disabilities may show more or less variability in their daily leg movement behavior than infants with typical development, resulting in a need for more or less data to accurately represent their typical daily behavior. Moreover, adults show different physical activity during weekday and weekend days [33,34]. As infants’ daily behavior highly relies on their caregivers, it is still not clear whether caregivers’ daily routine will affect infants’ behavior across the week. Moreover, the interaction between the infants and their siblings, who mostly go to school on weekdays, may also influence infants’ motor behavior.

Another big challenge conducting studies with infants with or at risk of developmental disabilities is the adherence level. Infants with or at risk of developmental disabilities tend to require a lot of time going to clinics or receiving early intervention. Having data collected from a long period of time will give researchers more reliable data, however, it will likely decrease the families’ willingness and ability to participate in the studies. We need to find a balance between getting reliable data and requiring a reasonable amount of time from the families. This may also help to shorten the data collection duration and develop a more efficient measurement protocol using wearable sensors.

This study provides a first step toward filling these gaps and accurately measuring movement behavior in infants with or at risk of developmental disabilities using wearable sensors. The purpose of this study is to determine (1) the minimum number of days of wearable sensor data needed to represent the typical daily leg movement behavior for infants at risk of developmental disabilities and (2) whether there is a difference between weekend days and weekdays for this group. The results of this paper will help to identify the balance between efficiently and accurately measuring infant leg movement behavior using wearable sensors.

## 2. Methods

### 2.1. Participants

Sixteen infants (8 females and 8 males) at risk of developmental disabilities within 2–14 months of age (mean (SD) = 250 ± 126 days, adjusted for prematurity as appropriate) participated in this study. Infants were recruited at the Children’s Hospital of Los Angeles Newborn Follow-Up Program. Inclusion criteria were infants who qualify for high-risk infant follow-up services in the State of California [35]. This represents a heterogeneous group that includes infants born with low birth weight, preterm birth, with complications at or after birth, etc. Thus, infants may be at different levels of developmental delay, which represents the typical distribution at clinical settings. Exclusion criteria were infants with unstable medical conditions. The characteristics of each infant’s birth information and anthropometric measurements are reported in Table 1.

### 2.2. Procedure

This research study was approved by the Institutional Review Board of the University of Southern California (HS-16-00170). A parent or legal guardian signed the informed consent form before their infant participated in the study. Data collection took place in the families’ homes.

#### 2.2.1. Wearable Sensor Data

On the first day of data collection, a researcher put a wearable sensor on each ankle of each infant. The sensors were attached using custom leg warmers with a pocket for each sensor. Each sensor weighed 22 g, measured 48.4 mm × 36.1 mm × 13.4 mm, and collected tri-axial accelerometer, gyroscope, and magnetometer data at 20 Hz (Opal sensor; APDM Inc., Portland, OR, USA). Parents were instructed to take the sensors off at bedtime, charge the sensors overnight, and put them back on each morning for 7 consecutive days. Sensors were labeled for the left or right leg. Parents filled out a daily survey describing whether each day was typical and recorded the time they put on or took off the sensors. Some families did not complete 7 consecutive days of data collection so we collected another week of data. However, many still did not complete 7 consecutive days of data collection. We decided to use 5 days of recorded data over a 7–14-day period in the final sample. The first 5 days that had at least 5 h of awake time data per day were included.

Infants’ leg movement data were stored on the sensors and downloaded after the last day of data collection using Motion Studio software (APDM Inc., Portland, OR, USA). Previously validated Matlab algorithms (The MathWorks Inc., Natick, MA, USA) were used to calculate leg movement characteristics. We described these algorithms [11,12] briefly here. The algorithm determined thresholds based on the resultant acceleration and angular velocity signals from across the day. Then the algorithm identified the start and end of each leg movement, based on threshold crossings, as described and validated in our previous publication. A single leg movement was defined by the algorithm each time the infant paused or changed the direction of the leg. This was validated against video observation identifying each leg movement. We first identified each leg movement made each day. Then we identified when infants were awake (infants generated more than 3 movements in 5 min). The daily leg movement rate was defined as the amount of leg movements per hour of awake time each day to normalize for different lengths of sensor wear and different amounts of nap times each day [11]. After we identified the beginning and end of each leg movement, we calculated its duration, average, and peak acceleration, as described in our previous publication [12].

#### 2.2.2. Anthropometrics, Videos, and Alberta Infant Motor Scale (AIMS)

During the first visit for each infant, we recorded 5-min video data of the infant’s spontaneous movements while wearing the sensors. These data are not presented in this paper. We also measured the infants’ anthropometrics (weight, head circumference, and body length) and assessed their motor development level using the Alberta Infant Motor Scale (AIMS) [36].

### 2.3. Outcome Measures

We analyzed 4 movement characteristics: average leg movement rate (the number of leg movements divided by the hours of awake time across a day), duration (the averaged leg movement duration of all leg movements across a day), average acceleration (the averaged average acceleration of all leg movements across a day), and peak acceleration per day (the averaged peak acceleration of all leg movements across a day). For each variable, we averaged the data for both legs for the following analysis.

### 2.4. Statistics

Out of the 16 infants, there were 5 who did not complete data collection for at least 5 h on 5 days, and thus were excluded from our analyses. This led to a total of 11 infants (6 females and 5 males) aged 4–17 months (mean (SD) = 336 ± 143 days) in the final analysis. The average wear time per day was 8.6 h (SD = 1.6). For comparing the average of 5 days to 1 day and the average of 2, 3, and 4 days, we analyzed data from 11 infants who finished at least 5 days of data collection and wore the sensors for at least 5 h each day on days that were typical days per caregiver report. For comparing weekend to weekdays, we included data from 8 infants who provided data on 2 weekend days.

The average of 5 days was used as the standard here. We chose this standard considering the available data we obtained here and our previous publication showing that the average of 2 days of leg movement data is similar to the average results from 5 days of data in infants with typical development. The average value of the first day, the average of the first 2 days, the average of the first 3 days, and the average of the first 4 days were compared with the standard. We also compared the average of the first 2 weekend days with the average of the first 2 weekdays.

We used the median value to measure the central tendency because of the small sample size and non-normal distribution. The difference and absolute difference between the standard and each measurement method (the average of the first 1/2/3/4 days) were calculated to determine the similarity between each pair of measurement methods. We also calculated the Spearman correlation coefficients to measure the magnitude of association between the standard and each method.

Bland–Altman plots were used to help determine the consistency between each pair of measurement methods (the average of the first 1/2/3/4 days compared to the standard) [37,38]. The limit of agreement and the confidence interval are shown in the b-e panels of each Bland–Altman figure. The proportional bias was checked to determine whether the level of difference was related to the magnitude of each variable. To compare the average of the first (1/2/3/4) days with the average of 5 days, we used the average of 5 days for the abscissa since it is the standard here. To compare the average of the first 2 weekdays and the average of the first 2 weekend days, we used the traditionally method (average of 2 weekdays and 2 weekend days as the abscissa). Bland–Altman plots were generated using Matlab R2019a.

We determined that the minimum number of days needed to represent the typical daily leg movement behavior for infants at risk of developmental disabilities needed to meet the following criteria: (1) the absolute difference for each variable is smaller than 10% of the standard, (2) the Spearman correlation coefficient for each variable is above 0.7, and (3) 90% of the data is within the limit of agreement (in the Bland–Altman plot) for each variable.

The same methods and analysis were used to compare the data from 2 weekend days and the first 2 weekdays. The difference between the data from weekend days and weekdays was compared with the standard (average of 5 days). We determined that there would be no need to adjust for weekend days if 90% of the data is within the limit of agreement (in Bland–Altman plot) for each variable. All statistical tests were performed using Matlab R2019a. Plots were generated using Matlab R2019a and SPSS software (Version 22; IBM Corporation, Armonk, NY, USA).

## 3. Results

### 3.1. Leg Movement Rate

**Figure 1:**
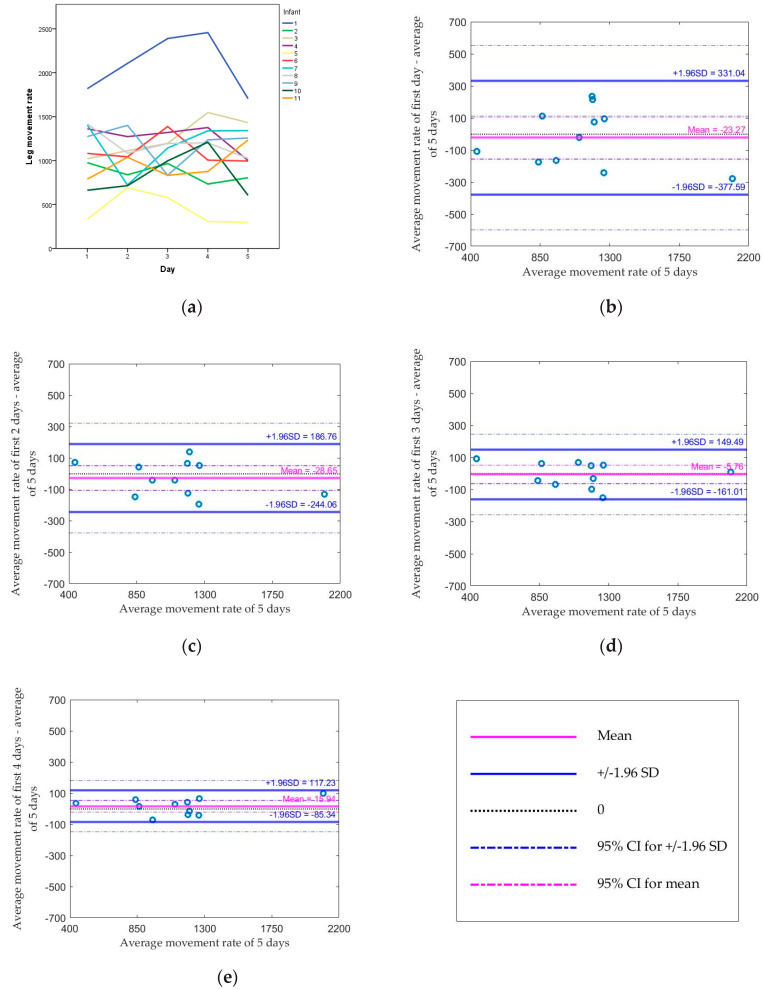
(**a**) Average leg movement rate (average leg movements per hour awake time) from the first day to the fifth day for each infant; Bland–Altman plots comparing (**b**) the first day, (**c**) the average of the first 2 days, (**d**) the average of the first 3 days, (**e**) the average of the first 4 days, and the average of 5 days for the average leg movement rate.

The average leg movement rates for all infants across five days are shown in Figure 1a. The range of leg movement rates for all infants was from 333 to 2193 movements per hour of awake time. Compared to the standard (average of 5 days), the median absolute difference dropped from 165 (using the first day) to 71 movements (using the average of the first two days), or from 13.9% to 6.0% of the standard. The median difference changed from −20 (using the first day) to −40 movements (using the average of the first two days) but the range became smaller. The Spearman correlation coefficient between each method and the standard increased from 0.80 by using the first day to 0.94 by using the average of the first two days (both *p* values < 0.05; Table 2). Based on Figure 1b–e, all values were within the limit of agreement using the average of the first two days. Moreover, the figure shows there was a proportional bias for the leg movement rate (the higher the individual’s leg movement rate was, the more this infant was different from the leg movement rate standard).

### 3.2. Duration

**Figure 2:**
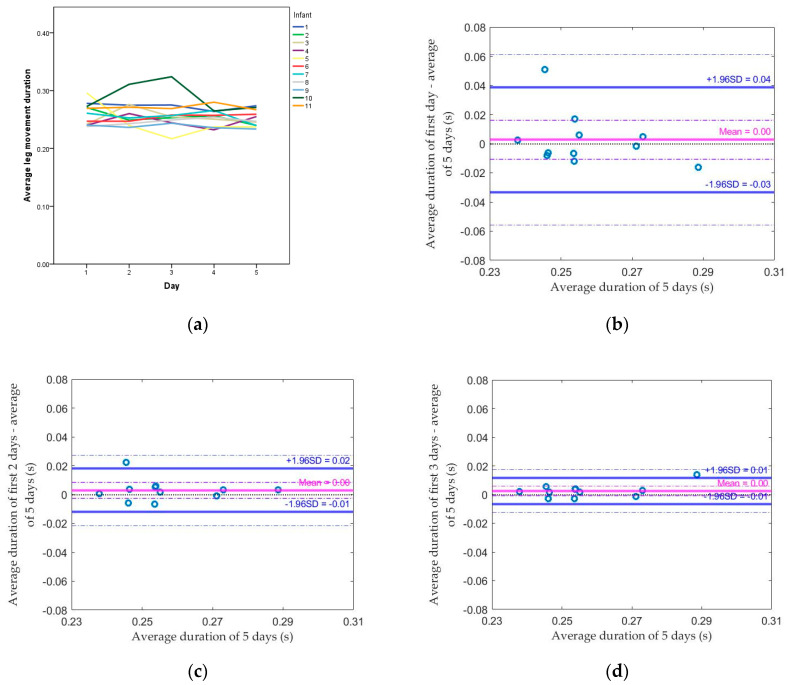

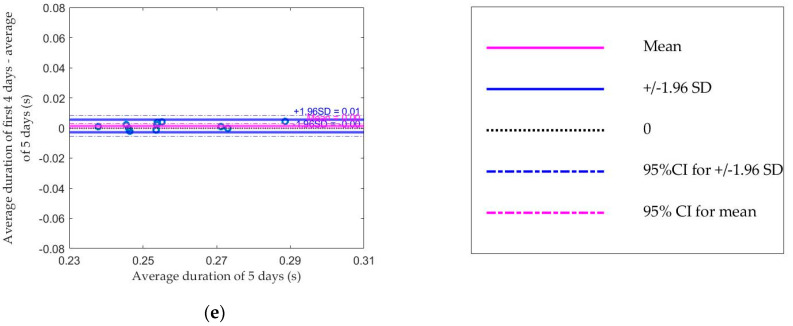
(**a**) Average leg movement duration from the first day to the fifth day for each infant; Bland–Altman plots comparing (**b**) the first day, (**c**) the average of the first 2 days, (**d**) the average of the first 3 days, (**e**) the average of the first 4 days, and the average of 5 days for the average leg movement duration.

The average leg movement durations across all daily leg movements for each infant across the five days are shown in Figure 2a. The range of average leg movement duration for all infants was 0.24–0.30 s. Compared to the standard (average of 5 days), the median absolute difference dropped from above 0.01 (using the first day) to below 0.01 (using the average of the first two days), or from 2.6% to 1.5% of the standard. The median difference slightly changed from using the first day to using the average of the first two days (both methods had a median of 0 but the average of 2 days had a smaller range). The Spearman correlation coefficient between each method and the standard increased from 0.50 by using the first day to 0.77 by using the average of the first two days (both *p* values < 0.05; Table 3). Based on Figure 2b–e, there was no proportional bias, which means the magnitude of difference was not related to the value of average leg movement duration. More than 90% of the data points were within the limit of agreement using the average of the first two days.

### 3.3. Average Acceleration

**Figure 3:**
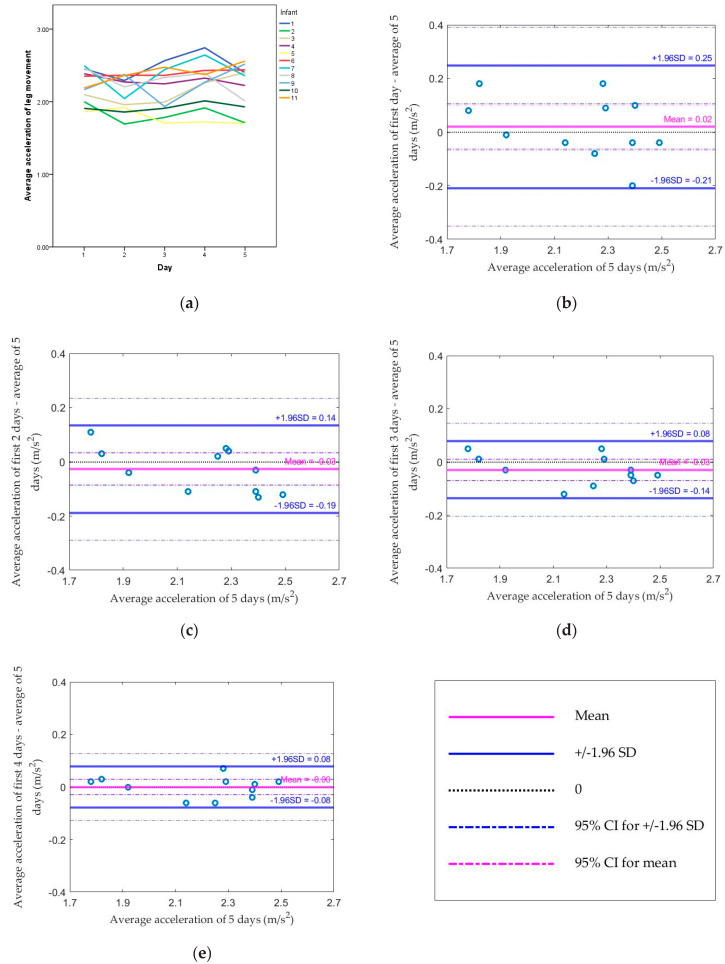
(**a**) Average acceleration of leg movement from the first day to the fifth day for each infant; Bland–Altman plots comparing (**b**) the first day, (**c**) the average of the first 2 days, (**d**) the average of the first 3 days, (**e**) the average of the first 4 days, and the average of 5 days for the average acceleration of leg movement.

The daily average of leg movement average accelerations across all daily leg movements for each infant across the five days are shown in Figure 3a. The range of average acceleration values for all infants was 1.781–2.513 m/s^2^. Compared to the standard (average of 5 days), the median absolute difference dropped from 0.085 (using the first day) to 0.052 (using the average of the first two days), or from 3.5% to 2.2% of the standard. The median difference also changed from −0.015 (using the first day) to −0.032 (using the average of the first two days). The Spearman correlation coefficient between each method and the standard changed from 0.85 by using the first day to 0.77 by using the average of the first two days (both *p* values < 0.05; Table 4). Based on Figure 3b–e, there was no proportional bias, which means the magnitude of difference was not related to the values of the average acceleration of leg movement. All values were within the limit of agreement using the average of the first two days.

### 3.4. Peak Acceleration

**Figure 4:**
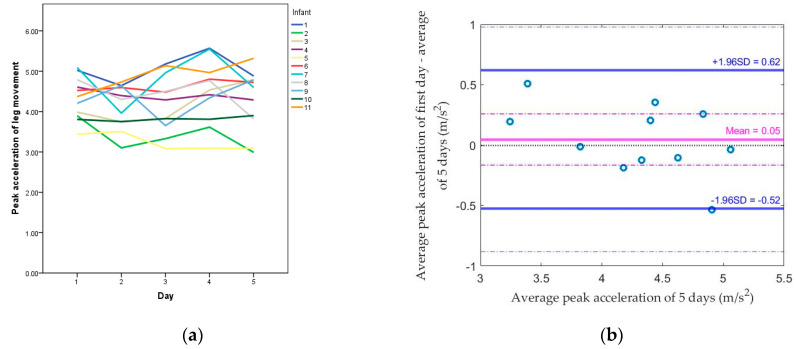

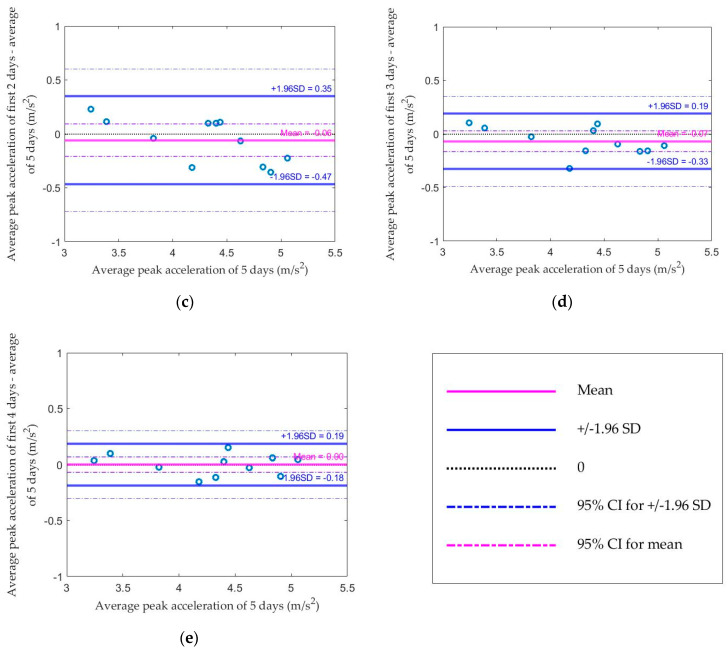
(**a**) Peak acceleration of leg movement from the first day to the fifth day for each infant; Bland–Altman plots comparing (**b**) the first day, (**c**) the average of the first 2 days, (**d**) the average of the first 3 days, (**e**) the average of the first 4 days, and the average of 5 days for the peak acceleration of leg movement.

The daily average of leg movement peak accelerations across all daily leg movements for each infant across the five days is shown in Figure 4a. The range of peak acceleration values for all infants was from 3.242 to 5.103 m/s^2^. Compared to the standard (average of 5 days), the median absolute difference dropped from 0.197 (using the first day) to 0.112 (using the average of the first two days), or from 4.5% to 2.6% of the standard. The median difference also changed from −0.013 (using the first day) to −0.040 (using the average of the first two days) with a smaller range. The Spearman correlation coefficient between each method and the standard increased from 0.85 by using the first day to 0.95 by using the average of the first two days (both *p* values < 0.05; Table 5). Based on Figure 4b–e, there was no proportional bias, which means the magnitude of difference was not related to the value of peak acceleration of leg movement. All values were within the limit of agreement using the average of the first two days.

### 3.5. Weekend Days Versus Weekdays

**Figure 5:**
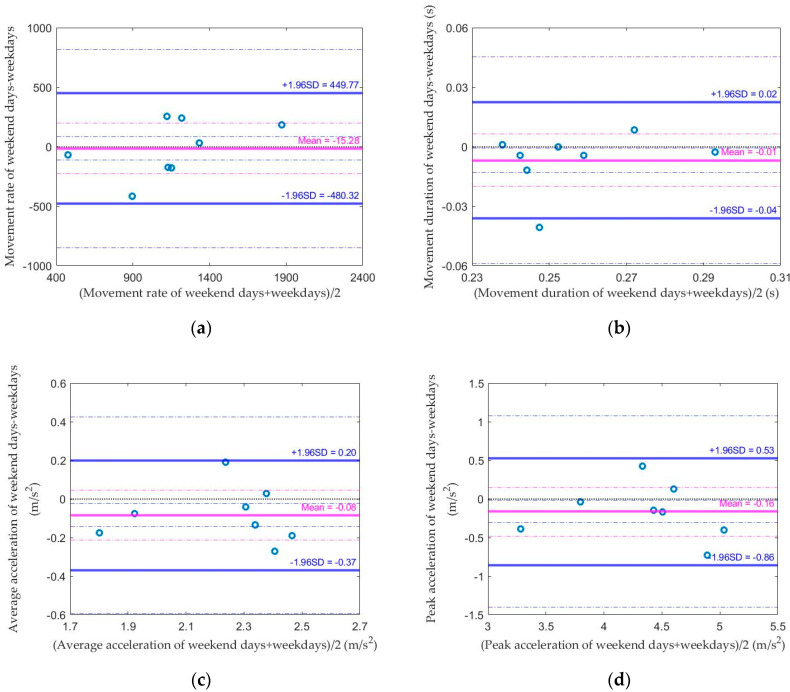

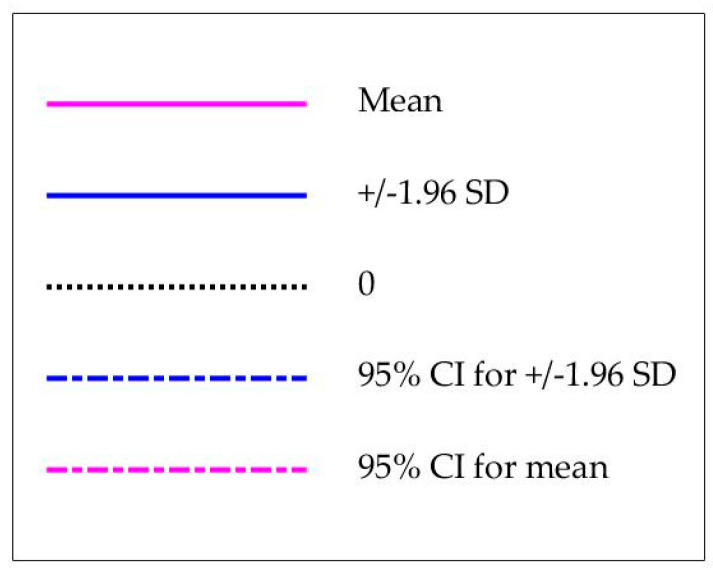
Bland–Altman plots comparing the average of the weekend days and the weekdays for the (**a**) average leg movement rate, (**b**) average leg movement duration, (**c**) average acceleration of leg movement, and (**d**) peak acceleration of leg movement.

As shown in Table 6, the average daily values for all variables on weekend days and weekdays were similar to each other. The median absolute difference between weekends and weekdays for leg movement rate, duration, average acceleration, and peak acceleration were 15.2%, 1.7% 6.8%, and 6.3% compared to each corresponding standard. The Spearman correlation coefficient for leg movement rate, duration, average acceleration, and peak acceleration between weekend days and weekdays all showed a moderate strength of correlation (0.64, 0.43, 0.52 and 0.67, respectively). Bland–Altman plots (Figure 5) show that most data points are within the limit of agreement (100%, 87.5%, 100% and 100%, respectively).

## 4. Discussion

The goal of this study was to recommend the minimum wear time for wearable sensors to accurately represent typical daily leg movement behavior while minimizing participant burden for infants at risk of developmental disabilities. We determined that 2 days of wearable sensors data is the best recommendation to represent the typical daily leg movement behavior for infants at risk of developmental disabilities. The average of 2 days provides a more accurate representation of typical behavior than a single day does. While more than 2 days of data will provide slightly greater reliability, it also increases participant burden. As there was not a large change from 2 days to 3, 4, or 5 days, 2 days seemed the best ‘balance’ between reliability and participant burden.

The Spearman correlation coefficients for each variable (leg movement rate, duration, average acceleration, and peak acceleration) were all above 0.7 after 2 days of data collection and the absolute differences all dropped below 10% of each standard (average value for 5 days). The magnitude of the median difference for all variables also dropped from 1 day to the average of 2 days except the leg movement rate. The increase of the magnitude of the median difference for the leg movement rate from 1 day to the average of 2 days is likely due to the variability of the data. However, the range of the difference, a more reliable method to look at this small sample size, decreased greatly from 1 day to the average of 2 days similar to the other variables. Bland–Altman plots also showed large reductions of limit of agreement size from day 1 to day 2 (28%–57%). Using more than 2 days did not change the results much and would be a greater burden for caregivers considering their already busy schedule for taking care of infants at risk for developmental disability. In fact, a previous study showed that extending the data collection requirement from 2 to 5 days led to missing days and half of the group being excluded from the final data sets for children with cerebral palsy [20]. Collecting data for a longer time will always improve the reliability. However, to consider the balance between participant burden and the amount of data collected and optimizing the efficiency of data collection, 2 days of data collection is our recommendation.

Assessing and quantifying infant movement behavior is challenging, especially for at-risk groups. Within this study, many of the infants had frequent health-care appointments, which affected their participation in the study. Some infants experienced changes in their health status and only wore the sensors for part of the 7 days or did not wear them at all. In these cases, we extended the data collection period for additional days; however, this did not always result in more data being collected.

The minimum wear-time recommended for the use of accelerometers to measure aspects of movement behavior has been described previously for infants and children with typical development. Similar to our finding that 2 days is recommended when measuring daily leg movement characteristics in infants with typical development [12], Pitchford et al. estimated at least 2 days and 12 h of data collection is needed to measure physical activity using accelerometers at the ankle for infants with typical development in the first year of life [31]. As age increases, previous literature suggests the minimum wear time to represent typical behavior gradually increases. For preschoolers (3–5 years), researchers recommended 2–4 days of data collection to measure their physical activity level [29,39,40]. For older children and teenagers, an even longer time (4–7 days) was recommended to achieve a reliable estimation of their physical activity level [41,42,43,44]. The need for longer data collection time may be related to differences across days in family or school schedules that may influence a student’s availability to participate in physical activities across days. However, one important note is that all of the above studies other than ours measured the intensity of physical activity instead of specific movement characteristics as we do. Compared to the typical accelerometers on the market, our sensors provide more detailed quantitative data (including tri-axial accelerometer and gyroscope data), which allows us to specifically capture movement quantity, duration, average acceleration, and peak acceleration of infant leg movements.

For infants and children with developmental disabilities, researchers have been using accelerometers to measure their physical activity levels. However, only in cerebral palsy have researchers gathered enough data to determine the minimum wear time needed to get a reliable result of their physical activity level. For children between 6 and 18 years old with cerebral palsy, 2–3 days of wear time are needed to get a reliable result [18,20]. For children with cerebral palsy between 2 and 5 years of age, 8, 6, and 2 days are needed for those with Gross Motor Function Classification System levels I, II, and III, respectively [21]. These previous studies show that age is an important factor to consider for recommending minimum wear times for wearable sensors. Our method to accurately measure infant behavior for at-risk groups is important considering the vital timing for early intervention. Our study suggests 2 days is the minimum wear time needed to represent typical daily leg movement behavior for infants at risk of developmental disabilities.

Originally, we expected that infants at risk of developmental disabilities might require less time to capture their typical behavior than infants with typical development. In the previous adult literature, at-risk groups have less day-to-day variability and require less days to represent their typical physical activity level than healthy groups. For example, 3–5 days (using an accelerometer) or 4–7 days (using a pedometer) are needed to measure the typical physical activity level for a healthy adult group [45,46,47,48,49]. A group of adults with a spinal cord injury only needed 2 days of activity monitoring to sufficiently represent their daily behavior [50]. We did not see a similar difference between typical and at-risk groups in our infant population. This may be due to the fact that adults have more day-to-day variability in terms of the physical activity level compared to infants, especially considering the weekend days. Infants, on the contrary, may have a more similar day to day schedule. Using the same wearable sensors, similar to the at-risk group here, we found 2 days of wear time on the ankle is recommended to represent typical leg movement behavior in infants with typical development [14].

Similar to infants with typical development, the difference between weekend days and weekdays was not significant in infants at risk of developmental disabilities in our study. The absolute difference between weekend days and weekdays for the leg movement rate, duration, average acceleration, and peak acceleration was 15.2%, 1.7%, 6.8%, and 6.3% of the corresponding standard. We analyzed the difference in leg movements on weekend days and weekdays considering that the caregiver and sibling’s different daily schedules across the week may influence the infant’s behavior. Adults and young students show different sedentary behavior on weekdays and weekends [33,39,48,51]. Based on the caregivers’ reports in our study, most families had similar daily schedules on weekdays and weekends. A few families went to parks or visited relatives during the weekend. Our results for infants at risk are similar to infants with typical development [14] and healthy preschoolers, where there is also no difference between weekdays and weekend days [14,52]. This indicates that a caregiver’s schedule across the week does not necessarily influence infant and young children’s movement behavior. We recommend it is not necessary to adjust for weekend days for the data collection for infants at risk of developmental disabilities.

Studies on wearable technologies have been increasing over the last decade, likely due to the relatively low cost of sensors and their high ecological feasibility for in-home/community data collection. With the decreasing cost and development of internet and remote platforms for data collection, sensors could become as widely used as mobile phones. They may be used to monitor daily behavior in real-time and provide contingent health care remotely or provide longitudinal data for research purposes [15]. For infants at risk of developmental disabilities, sensors could be used to monitor the infant’s dynamically changing movement behavior patterns and provide detailed information to customize early interventions, support their learning process, and optimize functional outcomes. For these reasons, wearable technology has the potential to change daily care and research in the future.

We acknowledge the small sample size and diverse ages and developmental levels of our sample. The results may not be generalized to infants with specific developmental disabilities, as some populations may have unique limb movement characteristics due to specific impairments. Our sample is, however, representative of infants in a high-risk infant follow up program. It represents a diverse group with different symptoms and developmental levels as encountered in clinics. Future studies might investigate specific developmental disabilities, ages, and/or developmental levels with a larger group. Some infants were excluded at the final analysis stage because of incomplete data collection. These infants may have different behavioral patterns compared with the current group, which may have introduced some bias towards our final results. However, this is the very first step to address the minimum wear time required for infants at risk of developmental disabilities. We did not look into how many hours is the minimum necessary to represent a single day. However, a previous study showed that the difference of reliability between using 3 h/day and using 10 h/day of accelerometer data is small [42]. We only collected data within a 2 week range for each infant, future studies looking at infant behavior across weeks and even months are needed to assess for behavior changes seasonally and longitudinally. Further, while we reported results from infants with typical development in a previous similar study [14], a control set of infants was not included in this study and we note that the duration of data collection periods was slightly different between the two studies. The results presented in this paper only refer to the wearable sensors positioned on the ankle. Future studies need to investigate whether the result would be different if the sensors are placed on the wrist or trunk, considering the location of sensors will also affect the variability of data [52]. Lastly, we did not specifically consider external motion during the data collection, for example passive movement of the infants’ limbs generated by caregivers or other sources [53,54]. We assumed that these factors would be consistent across days, which may or may not be true. Further, our algorithm effectively filters out some external motion by analyzing both acceleration and angular velocity signals at the same time. For example, the presence of an acceleration signal with a magnitude above the threshold but without the presence of angular velocity above the threshold is not likely due to infant limb movement, but instead is likely attributable to the background acceleration of the infant being carried or in a mechanical swing [11].

## 5. Conclusions

After checking the similarity of values of leg movement characteristics from various numbers of days of data collection, we recommend using 2 days of wearable sensor data to represent typical daily leg movement behavior in infants at risk of developmental disabilities. Moreover, there was no significant difference between weekend days and weekdays. These results provide guidance for future research and clinical assessment of full day infant motor behaviors in infants at risk for developmental disabilities using wearable sensors. While we acknowledge the small sample size and limited generalizability of our results, this work represents a foundational step for early detection and description of infants at risk using wearable technologies.

## Figures and Tables

**Table 1:** Participant’s health status, anthropometric measurements, and motor developmental level.

Infant	Health Status Summary	Gestational Age at Birth (Weeks)	Chronological Age (Days)	Adjusted Age (Days)	Gender (M = Male, F = Female)	Weight (kg)	Length (cm)	Head Circumference (cm)	AIMS (Total)	AIMS (Percentile)	Included for Final Analysis?
1	preterm, short gut syndrome	25	495	395	F	8.22	78.0	42.4	24	<5	Yes
2	preterm	30	138	74	F	4.81	54.0	37.8	7	10–25	Yes
3	preterm	26	234	137	M	6.07	59.5	41.8	14	10–25	Yes
4	preterm	24	441	333	F	6.49	68.5	42.0	49	25–50	Yes
5	preterm, intraventricular hemorrhage	30	182	112	M	5.88	55.5	35.8	8	5–10	Yes
6	preterm	31	263	202	M	8.31	67.0	43.5	20	5–10	Yes
7	preterm, on oxygen	23	339	224	F	7.77	63.0	41.0	12	<5	Yes
8	preterm	25	536	433	F	9.88	70.0	46.0	52	<5	Yes
9	preterm	25	536	433	M	8.39	69.0	46.5	52	<5	Yes
10	congenital heart defects	41	262	NA	M	8.02	67.0	45.0	33	10–25	Yes
11	preterm, gets breathing treatment	35	273	239	F	9.22	68.0	44.0	40	50–75	Yes
12	preterm, gastroparesis	28	498	417	F	7.49	68.5	45.2	58	75–90	No
13	preterm, on ventilator and feeding tube	33	147	114	M	6.46	62.5	41.0	13	25–50	No
14	preterm	29	306	233	M	9.07	66.0	46.0	16	<5	No
15	omphalocele, Beckwith- Weidmann syndrome	36	301	-	F	11.47	72.0	48.0	27	<5	No
16	DiGeorge syndrome	40	85	-	M	5.01	51	40	8	10–25	No

AIMS = Alberta Infant Motor Scale.

**Table 2:** Average values for leg movement rate.

Parameter	Median (Range)	Median of Absolute Difference (Range) Compared with Average for 5 d	Median of Difference (Range) Compared with Average for 5 d	Spearman Correlation for Each Method Compared with Average for 5 d
First day (d)	1082 (333, 1818)	165 (20, 277)	−20 (−277, 234)	0.80
Average for first 2 d	1064 (512, 1962)	71 (40, 195)	−40 (−195, 137)	0.94
Average for first 3 d	1110 (534, 2105)	63 (9, 152)	9 (−152, 93)	0.85
Average for first 4 d	1152 (477, 2193)	40 (14, 97)	27 (−70, 97)	0.92
Average for first 5 d	1185 (441, 2095)	-	-	-

**Table 3:** Average values for the duration of leg movement.

Parameter	Median (Range)	Median of Absolute Difference (Range) Compared with Average for 5 d	Median of Difference (Range) Compared with Average for 5 d	Spearman Correlation for Each Method Compared with Average for 5 d
First day (d)	0.26 (0.24, 0.3)	0.01 (0, 0.05)	0 (−0.02, 0.05)	0.50
Average for first 2 d	0.26 (0.24, 0.29)	<0.01 (0, 0.02)	0 (−0.01, 0.02)	0.77
Average for first 3 d	0.26 (0.24, 0.3)	<0.01 (0, 0.01)	0 (0, 0.01)	0.92
Average for first 4 d	0.26 (0.24, 0.29)	<0.01 (0, 0.01)	0 (0, 0.01)	0.96
Average for first 5 d	0.25 (0.24, 0.29)	-	-	-

**Table 4:** Average acceleration of leg movement.

Parameter	Median (Range)	Median of Absolute Difference (Range) Compared with Average for 5 d	Median of Difference (Range) Compared with Average for 5 d	Spearman Correlation for Each Method Compared with Average for 5 d
First day (d)	2.190 (1.863, 2.496)	0.085 (0.015, 0.202)	−0.015 (−0.202, 0.180)	0.85
Average for first 2 d	2.270 (1.846, 2.372)	0.052 (0.019, 0.126)	−0.032 (−0.126, 0.109)	0.77
Average for first 3 d	2.301 (1.825, 2.436)	0.050 (0.005, 0.126)	−0.031 (−0.126, 0.053)	0.91
Average for first 4 d	2.307 (1.802, 2.513)	0.023 (0.001, 0.068)	0.010 (−0.067, 0.068)	0.98
Average for first 5 d	2.279 (1.781, 2.490)	-	-	-

**Table 5:** Peak acceleration of leg movement.

Parameter	Median (Range)	Median of Absolute Difference (Range) Compared with Average for 5 d	Median of Difference (Range) Compared with Average for 5 d	Spearman Correlation for Each Method Compared with Average for 5 d
First day (d)	4.371 (3.439, 5.091)	0.197 (0.013, 0.535)	−0.013 (−0.535, 0.509)	0.85
Average for first 2 d	4.501 (3.474, 4.833)	0.112 (0.040, 0.354)	−0.040 (−0.354, 0.232)	0.95
Average for first 3 d	4.430 (3.344, 4.948)	0.102 (0.024, 0.323)	−0.092 (−0.323, 0.102)	1.00
Average for first 4 d	4.427(3.281, 5.103)	0.059 (0.021, 0.154)	0.028 (−0.152, 0.154)	0.99
Average for first 5 d	4.400 (3.242, 5.059)	-	-	-

**Table 6:** Leg movement rate, duration, average acceleration, and peak acceleration of leg movements on weekends versus weekdays.

Parameter	Median (Minimum, Maximum) on:		Median Absolute Difference between Weekend Days and Weekdays (Range)	Median Difference between Weekend Days and Weekdays (Range)	Spearman Correlation for Leg Movement Rate, Duration, Average Acceleration, and Peak Acceleration between Weekend Days and Weekdays
	Weekend Days	Weekdays			
Leg Movement Rate	1159 (512, 1778)	1157 (443, 1962)	180 (31, 415)	−20 (−415, 257)	0.64
Duration: seconds	0.26 (0.24, 0.29)	0.25 (0.23, 0.29)	<0.01 (0, 0.04)	<0.01(−0.04, 0.01)	0.43
Average acceleration: m/s^2^	2.346 (1.890, 2.561)	2.278 (1.714, 2.391)	0.156 (0.027, 0.272)	−0.106 (−0.272, 0.190)	0.52
Peak acceleration: m/s^2^	4.520 (3.474, 5.255)	4.475 (3.087, 4.833)	0.277 (0.039, 0.728)	−0.157 (−0.728, 0.427)	0.67
